# Response efficacy of PD-1 and PD-L1 inhibitors in salivary gland cancers: a systematic review and meta-analysis

**DOI:** 10.3389/fonc.2025.1534343

**Published:** 2025-04-02

**Authors:** Zahra Babamohamadi, Amirreza Taherkhani, Ali Yousefzadeh, Ali Moradi, Marziyeh Heidari, Zahra Sadat Hoseini Nasab, Seyedeh Mona Haghi, Ayda Afkhami, Mohaddeseh Belbasi, Masoud Noroozi, Niloofar Deravi

**Affiliations:** ^1^ Hearing Disorders Research Center, Loghman Hakim Hospital, Shahid Beheshti University of Medical Sciences, Tehran, Iran; ^2^ Student Research Committee, School of Medicine, Shahid Beheshti University of Medical Sciences, Tehran, Iran; ^3^ Center for Translational Medicine, Semmelweis University, Budapest, Hungary; ^4^ Faculty of Medicine, Hormozgan University of Medical Sciences, Bandar Abbas, Iran; ^5^ School of Medicine, Mashhad University of Medical Science, Mashhad, Iran; ^6^ Student Research Committee, School of Pharmacy, Mashhad University of Medical Science, Mashhad, Iran; ^7^ Student Research Committee, Tabriz University of Medical Sciences, Tabriz, Iran; ^8^ Students Research Committee, School of Pharmacy, Zanjan University of Medical Sciences, Zanjan, Iran; ^9^ Department of Biomedical Engineering, Faculty of Engineering, University of Isfahan, Isfahan, Iran

**Keywords:** salivary gland cancer, PD-L1 inhibitor, PD-1 inhibitor, meta-analysis, check point inhibitor

## Abstract

**Background:**

Salivary gland carcinoma (SGC) is an infrequent malignancy characterized by various pathological subtypes. Immune checkpoint inhibitors (ICIs) have emerged as promising therapeutic strategies for several cancers. Evidence suggests that ICIs may be effective against rare neoplasms, including SGC. This meta-analysis evaluated the efficacy of programmed death-1 (PD-1) and programmed death ligand 1 (PD-L1) inhibitors in treating salivary gland cancers.

**Method:**

A thorough search was conducted in PubMed, Scopus, and Google Scholar databases up to 24 February 2024. The title, abstract, and full text of related articles were extracted and screened, and the quality of the included articles was assessed. The data extracted were then analyzed. The research protocol of this systematic review and meta-analysis was registered on the PROSPERO website.

**Results:**

Altogether, a total of five cohort studies and three randomized controlled trials (RCTs) with a total population of 532 were included in these meta-analyses. These studies were conducted in the USA, Japan, France, and China. The average age of the patients was between 53 and 67 years. Our analyses showed an increase in progression-free survival in the cohort studies and RCTs, and the pooled effect is 1.12 (95% CI 1–1.25) and 1.14 (95% CI 1.07–1.20), respectively, in patients with SGC who received PD-1 and PD-L1 inhibitors.

**Conclusion:**

This meta-analysis suggests that PD-1 and PD-L1 inhibitors in patients with salivary gland cancer can significantly increase progression-free survival. Due to the high heterogeneity of the studies, more RCTs with a larger sample size are required to prove the association.

## Introduction

Salivary gland carcinoma (SGC) is a less common form of malignancy compared to other types of head and neck cancers. The occurrence rate of SGC is estimated to be 25–30 individuals per one million. SGC makes up less than 5% of all malignancies and approximately 5% of all head and neck cancers (1, 2). Regardless of its histological subtype, the curative treatment involves a surgical resection, with or without postoperative adjuvant radiation therapy (1, 3, 4). Surgical operation is the fundamental treatment for salivary gland cancers due to their resistance to chemotherapy and radiotherapy. A multidisciplinary approach is often necessary for appropriate management (5, 6). Complete resection and postoperative irradiation are recommended based on histological findings, especially if there is a positive surgical margin. In cases of recurrence, metastasis, or unresectable tumors, chemoradiotherapy or chemotherapy may be considered as initial treatment (3, 4, 7). These treatments all have a common approach in activating the body’s immune system to eliminate cancer cells (8, 9). Despite the immune system being a key player in fighting against tumors, cancer cells originate from the patient’s cells and maintain various natural defense mechanisms that can prevent the immune-mediated destruction of tumors (10). Programmed death-1 (PD-1) is an immune checkpoint and a co-stimulatory molecule. This regulatory protein is found on T cells and pro-B cells, playing a crucial role in the negative regulation of T-cell activation and immune responses (11). The interaction between programmed death ligand 1 (PD-L1) on tumor cells and PD-1 on tumor-specific T cells leads to the suppression of T-cell cytotoxic activity (1, 12, 13). Consequently, the activated PD-1/PD-L1 pathway enables tumor cells to escape detection by the immune checkpoint system (9). Furthermore, reports have indicated that an increased expression of PD-L1 is closely associated with poor prognosis in many different malignancies, such as carcinomas of the kidney, esophagus, stomach, pancreas, and breast and malignant melanoma (14–19). However, the relationship between PD-L1 expression in SGCs and clinicopathological behavior is still unknown. To the best of our knowledge, this meta-analysis aims to investigate the association of response efficacy between PD-1 and PD-L1 inhibitors in salivary gland cancers for the first time.

## Methods

This systematic review and meta-analysis aims to investigate the efficacy of PD-1 and PD-L1 inhibitors in treating salivary gland cancers. Our methodology follows the Preferred Reporting Items for Systematic Reviews and Meta-analyses (PRISMA) guidelines (20). The research protocol of this systematic review and meta-analysis was registered on the PROSPERO website.

### Literature search

A comprehensive literature review was conducted until 24 February 2024, to identify pertinent articles from PubMed, Scopus, and Google Scholar databases. In the search strategy, two primary subgroups of keywords and Medical Subject Headings (MeSH) were utilized in **Table 1**. The first subgroup comprised terms associated with PD-1 and PD-L1, while the second included terms about salivary cancer. The “AND” operator combined the subgroups without imposing any limitations on the date, publication type, or language. The search methodology was modified based on the query format specific to each database. We thoroughly examined the reference lists of relevant systematic reviews to mitigate the potential for overlooking pertinent scholarly articles. We incorporated studies that met the criteria for inclusion in our research. The reviewers independently carried out all the steps, and any disagreements were resolved through deliberation.

**Table 1:** Search strategy.

Search engine	Search strategy	Additional filters	Total results
PubMed	Search: (((((((((((pdl1[Title/Abstract]) OR (pd1[Title/Abstract])) OR (nivolumab[Title/Abstract])) OR (pembrolizumab[Title/Abstract])) OR (atezolizumab[Title/Abstract])) OR (avelumab[Title/Abstract])) OR (darvalumab[Title/Abstract])) OR (cemiplimab[Title/Abstract])) OR (dostarlimab[Title/Abstract])) OR (retifanlimab[Title/Abstract])) OR (toripalimab[Title/Abstract])) AND (salivary gland(s)[Title/Abstract]) (“pdl1”[Title/Abstract] OR “pd1”[Title/Abstract] OR “nivolumab”[Title/Abstract] OR “pembrolizumab”[Title/Abstract] OR “atezolizumab”[Title/Abstract] OR “avelumab”[Title/Abstract] OR “cemiplimab”[Title/Abstract] OR “dostarlimab”[Title/Abstract] OR “retifanlimab”[Title/Abstract] OR “toripalimab”[Title/Abstract]) AND “salivary gland(s)”[Title/Abstract]	24 February 2024	121
Scopus	(TITLE-ABS-KEY (salivary AND gland(s)) AND TITLE-ABS-KEY (pdl1) OR TITLE-ABS-KEY (pd1) OR TITLE-ABS-KEY (nivolumab) OR TITLE-ABS-KEY (pembrolizumab) OR TITLE-ABS-KEY (atezolizumab) OR TITLE-ABS-KEY (avelumab) OR TITLE-ABS-KEY (darvalumab) OR TITLE-ABS-KEY (cemiplimab) OR TITLE-ABS-KEY (dostarlimab) OR TITLE-ABS-KEY (retifanlimab) OR TITLE-ABS-KEY (toripalimab))	24 February 2024	194
Google Scholar	With all of the words: salivary gland(s) pdl1 pd1 nivolumab pembrolizumab atezolizumab avelumab darvalumab cemiplimab dostalimab retifanlimab toripalimab	24 February 2024	26

### Criteria for selecting studies

For studies to be included in this meta-analysis, they must satisfy the following criteria: 1) Observational methodology was employed to mitigate the potential confounding influence of any intervention. 2) The primary objective was to evaluate the effectiveness of particular immune checkpoint inhibitors (PD-1 and PD-L1) in treating salivary gland cancer. The study sample comprised individuals who had been diagnosed with salivary gland cancer and had undergone treatment with either PD-1 or PD-L1. 4) The definitions of salivary gland cancer were reported based on the study design. The study outlined the efficacy of PD-1 and PD-L1 in treating the salivary gland, with a particular focus on reducing mortality. 5) Excluded from consideration were studies that employed alternative methodologies, were conducted on animal models, or encompassed different types of cancer.

### Data extraction and study quality assessment

The eligibility for inclusion in this meta-analysis was determined by two independent reviewers who assessed the title and abstract of each study. Excluded were studies that did not meet our criteria. All the remaining studies were thoroughly examined, and only those that met the criteria were included in the data extraction process. Subsequently, the subsequent items were acquired for extraction in four distinct sets: 1) characteristics of the study, such as the authors, location, year, and type of study; 2) factors specific to the patients, including the criteria for participation, type of immune checkpoint inhibitors, and cancer type; 3) the study’s design including the number of participants, sampling method, period, and the definition of salivary cancer; and 4) outcomes including the efficacy of PD-1 and PD-L1 and the reduction in mortality and morbidity of salivary gland cancer. The critical appraisal checklists for cohort, case–control, and analytical cross-sectional studies, developed by the Joanna Briggs Institute (JBI), were utilized by two reviewers, as mentioned earlier (JBI, 2021). If there were any inconsistencies, a third author was involved in the process.

### Statistical analysis

Data analysis was conducted using STATA 13.1 software developed by StataCorp LP in College Station, TX, USA. The outcomes of the cohort studies were expressed as pooled odds ratios (ORs) with 95% confidence intervals and visually depicted in a forest plot. Meanwhile, RCT results were indicated using progression-free survival (PFS) values with 95% confidence intervals, also visually presented in a forest plot. The *I* ^2^ statistic was employed to assess heterogeneity among the eligible studies (21). The random-effects model was used when significant heterogeneity was observed (*I* ^2^ > 50%) (22). In addition, a sensitivity analysis was also performed by selectively excluding one study at a time and repeating the meta-analysis. This allowed us to guarantee the reliability of our results. We visually examined funnel plot symmetry and conducted Egger’s regression analysis to examine the possibility of publication bias (23).

## Results

### Study selection

**Figure 1:**
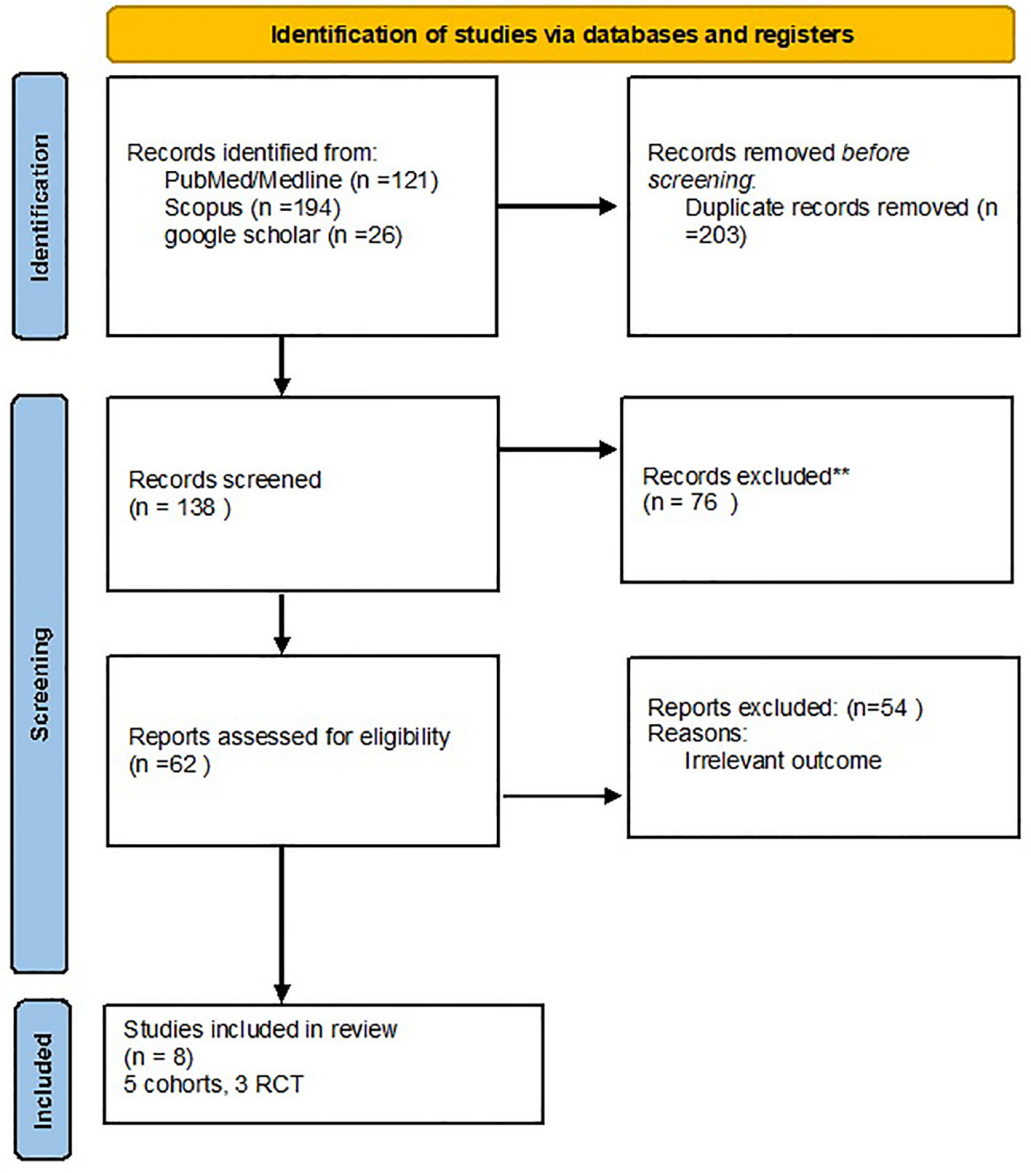
PRISMA flow diagram for the current systematic review and meta-analysis.

After searching in the PubMed, Scopus, and Google Scholar databases, a total of 341 articles were obtained, and 203 duplicates were removed. After reviewing the title and abstract screening, 62 studies remained. The final review included eight articles with full-text results, and articles with unrelated data were excluded (**Figure 1**).

### Baseline characteristics

The eight articles, involving a total of 532 patients, were reviewed. Of these eight studies, five were cohort studies, and three were RCTs. These studies were conducted in the USA, Japan, France, and China. The average age of the patients was between 53 and 67 years. Most of the articles discussed the survival and outcome of treatment of metastatic or advanced levels of salivary gland carcinoma with nivolumab (240 mg/2 weeks or 3 mg/kg/2 weeks), pembrolizumab (200 mg/3 weeks or 10 mg/kg/2 weeks), and ipilimumab (1 mg/kg/6 weeks) (**Table 2**).

**Table 2:** Summary of the included studies.

Author/reference	Year	Country	Study design	Participants	Sex (female)	Mean age	Intervention
Kazuki Hashimoto et al. (24)	2022	Japan	Cohort	36 patients with SGC	12 (33.3%)	67	Nivolumab (240 mg/body, once every 2 weeks) or pembrolizumab (200 mg/body, once every 3 weeks).
Chae et al. (25)	2023	China	RCT	19 patients with SGC	10 (52.6%)	55.2	Nivolumab (240 mg intravenously every 2 weeks for 16 weeks, then 480 mg every 4 weeks) with ipilimumab (1 mg/kg intravenously every 6 weeks).
Roger B. Cohen et al. (26)	2018	USA	Cohort	26 patients with SGC	3 (22%)	57	Pembrolizumab 10 mg/kg every 2 weeks for ≥2 years or until confirmed disease progression or unacceptable toxicity.
Jérôme Fayette (27)	2023	France	RCT	46 patients with ACC and 52 patients without ACC	46 (46%)	59/63	Nivolumab was administered as a 60 min ( ± 5 min) intravenous infusion at a fixed dose of 3 mg/kg on D1 and D15 of each 28-day cycle. All eligible patients received nivolumab treatment until disease progression or for a maximum of 12 cycles.
Yoshiaki Nagatani et al. (28)	2023	USA	RCT	24 patients with SGC	8 (33%)	65.5	Nivolumab 240 mg/body was administered intravenously every 2 weeks until progression or unacceptable toxicity.
Fayette (29)	2019	France	Cohort	98 patients with SGC 46 ACC and 52 non-ACC	43 (43.9%)	61	Received nivolumab 3 mg/kg IV, every 2 weeks for a maximum of 12 months.
Caroline Even (30)	2022	France	Cohort	109 patients with SGC	55 (50.5%)	53.3	Pembrolizumab 200 mg was administered as an intravenous infusion once every 3 weeks for up to 35 cycles (approximately 2 years) or until disease progression, unacceptable toxicity. Patients who attained complete response (CR) were permitted to stop pembrolizumab treatment after receiving at least eight treatment cycles.
Kazutomo Niwa et al. (31)	2020	Japan	Cohort	24 patients with SGC	5 (21%)	56	Nivolumab (240 mg) was administered every 2 weeks.

### Meta-analysis

**Figure 2:**
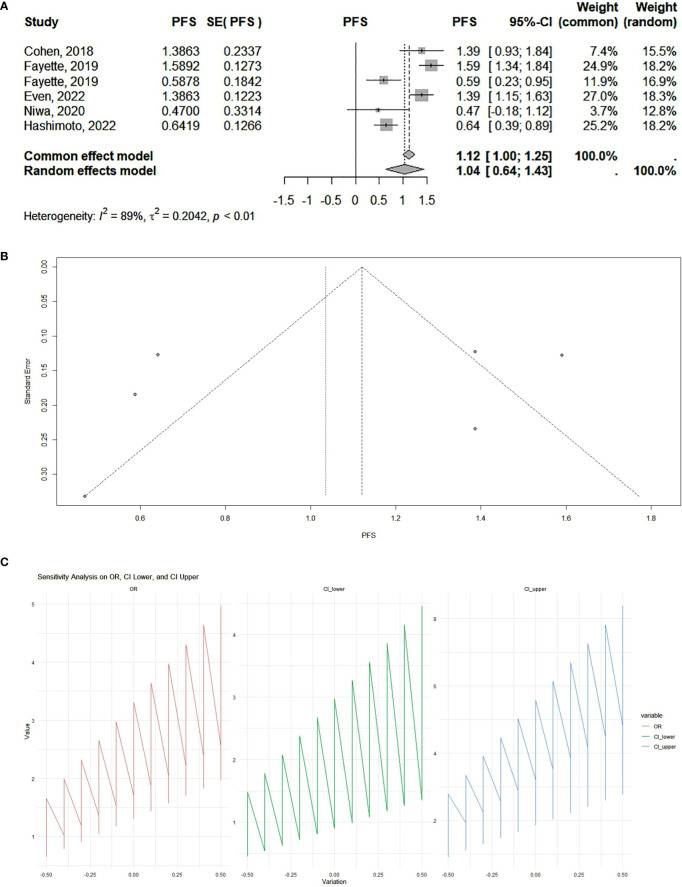
**(A)** Cohort studies: forest plot. The overall summary effect under the fixed-effects model is 1.12 (95% confidence interval: 1.00, 1.25). The overall summary effect under the random-effects model is 1.04 (95% CI: 0.64, 1.43). High heterogeneity is indicated (I² = 89%), reflecting significant variability in effect sizes among the studies. **(B)** Cohort studies: funnel plot. The presented funnel plot shows some degree of asymmetry, indicating possible publication bias or heterogeneity among the studies. **(C)** Cohort studies: sensitivity analysis. The sensitivity analysis plots demonstrate how the odds ratios (ORs) and their confidence intervals (CI) change with variations applied. The OR values and confidence intervals systematically vary across the implemented changes, indicating the robustness of the meta-analysis results. Large fluctuations in the lines may indicate sensitivity to specific studies or assumptions, while smaller fluctuations suggest robustness.

**Figure 3:**
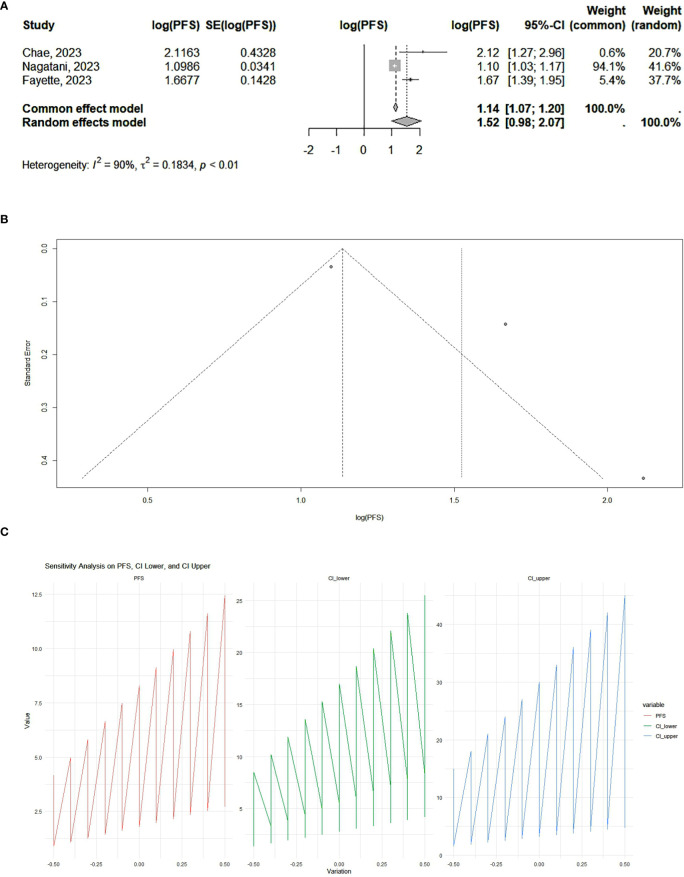
**(A)** RCT studies: forest plot. The overall summary effect under the fixed-effects model is 1.14 (95% CI: 1.07, 1.20). The overall summary effect under the random-effects model is 1.52 (95% CI: 0.98, 2.07). High heterogeneity is indicated (I² = 90%), reflecting significant variability in effect sizes among the studies. **(B)** RCT studies: funnel plot. The presented funnel plot shows some degree of asymmetry, suggesting potential biases. **(C)** RCT studies: sensitivity analysis. The sensitivity analysis plots illustrate how progression-free survival (PFS) and its confidence Intervals change with variations applied. The PFS values and confidence intervals systematically vary across the implemented changes, indicating the robustness of the meta-analysis results. Large fluctuations may indicate sensitivity to specific studies or assumptions, while smaller fluctuations suggest robustness.

Our results showed that PD-1 and PD-L1 inhibitors can significantly increase progression-free survival. According to the analysis, the pooled effect is 1.12 (95% CI 1.00–1.25) for cohort studies (**Figure 2**) and 1.14 (95% CI 1.07–1.20) for RCTs (**Figure 3**). The presented funnel plot shows some degree of asymmetry, indicating possible publication bias or heterogeneity among the studies. According to our sensitivity analysis, no study was removed from the meta-analysis.

## Discussion

This meta-analysis, with a total population of 532, revealed that the progression-free survival of salivary gland cancer patients can increase significantly following treatment with PD-1 and PD-L1 inhibitors. Considering both randomized controlled trials and cohort studies, the study results underscore that progression-free survival can increase significantly, emphasizing the potential therapeutic benefits of PD-1 and PD-L1 inhibitors for salivary gland cancer patients.

Hashimoto et al. (24) conducted a retrospective non-interventional study in Japan to assess the efficacy of PD-1 inhibitor therapeutic properties in recurrent/metastatic salivary gland cancer. From March 2017 to March 2021, they studied 36 patients who received nivolumab or pembrolizumab for recurrent/metastatic salivary gland cancer. They analyzed the overall survival (OS) as the timeline from the starting point of PD-1 inhibitor therapy to mortality from any cause. Applying the Response Evaluation Criteria in Solid Tumors version 1.1, they evaluated the best overall response (BOR), objective response rate (ORR), and disease control rate (DCR). In addition, they analyzed the PFS as the timeline from the starting point of PD-1 inhibitor therapy to the diagnosis point of disease progression or death. To add more information, they immunohistochemically analyzed the mismatch repair (MMR) proteins and expression of PD-L1. They found that patients with histopathology of SDC and poorly differentiated carcinoma had a positive PD-L1 expression and achieved a complete or partial response. They found no association between the MMR protein expression and the efficacy of PD-1 inhibitors. In a nutshell, the findings of their study revealed that limited patients may respond to the treatment option, achieving long-term disease control. The results of their study highlighted the potential therapeutic benefits in patients with salivary gland cancer who had positive PD-L1 expression in their profile (24).

Due to the limited therapeutic options for metastatic or unresectable salivary gland carcinoma, Cohen et al. (26) designed a multicohort, non-randomized phase Ib trial to evaluate the safety and efficacy of pembrolizumab in PD-L1-positive salivary gland carcinoma patients. They studied 26 patients with recurrent or metastatic salivary gland carcinoma with positive expression of PD-L1, who experienced treatment failure with systemic therapies. The median age of the patients was 57 years, 88% of whom were men. According to the study results, the objective response rate was 12% after a median follow-up of 20 months. Therapy-related adverse effects were observed in 85% of the patients, and in more than 15% of the cases, the adverse effects included decreased appetite, diarrhea, fatigue, and pruritus. In a nutshell, the findings of the study highlighted a promising antitumor property with manageable safety for advanced cases of PD-L1-positive salivary gland carcinoma (26).

According to a study, Fayette et al. (29) designed a phase II, multicenter non-randomized trial to assess nivolumab monotherapy in two cohorts of recurrent/metastatic head and neck salivary gland carcinoma (SGCHD) cases. The clinical observations indicated that SGCHD can include adenoid cystic carcinoma (ACC), which is a cold tumor with the absence of PD-L1, and non-adenoid cystic carcinoma (non-ACC). Unfortunately, standard systemic therapy for recurrent or metastatic cases had not been established previously. They included recurrent/metastatic cases of SGCHD who had not responded to local treatment and their disease progression over the last 6 months was confirmed. The enrolled cases received intravenous nivolumab 3 mg/kg, every 2 weeks with a maximum of 12 months. Forty-six ACC patients and 52 non-ACC cases with a median age of 61 years were included in this study. In their study, the median PFS was 4.9 months in ACC patients and 1.8 months in non-ACC patients. Adverse events, including hyper- or hypothyroidism, asthenia, pruritus, rash, and diarrhea, were also observed. In conclusion, although limited efficacy of nivolumab was observed in recurrent/metastatic cases of head and neck salivary gland carcinoma, it is interesting to evaluate nivolumab in combination with other therapeutic options (29).

Even et al. (30) researched advanced cases of salivary gland carcinoma in the phase 2 KEYNOTE-158 study to evaluate pembrolizumab monotherapy. They included 109 patients with histological or cytological confirmation for advanced salivary gland carcinoma who had a history of failure or intolerance to conventional therapy. Enrolled patients with 53.3 months of median follow-up received pembrolizumab monotherapy, irrespective of tumor PD-L1 expression. The objective response rate was 4.6% among all patients, 10.7% in PD-L1-positive cases, and 2.6% in PD-L1-negative patients. The findings were concurrent with 4 months of median progression-free survival and 21.1 months of overall survival. Also, therapeutic adverse effects were observed in 75.2% of patients, and 22% of cases experienced immune-mediated adverse events. In a nutshell, the study underscores the therapeutic benefits of pembrolizumab monotherapy in a small subset of cases with advanced salivary gland carcinoma and shows a manageable safety profile for the therapeutic option (30).

To expand our understanding of immune checkpoint inhibitors (ICIs) in SGC, Niwa et al. (31) designed a multicenter retrospective study to evaluate the efficacy and safety of nivolumab in recurrent/metastatic SGC patients. They enrolled 24 patients with the most common pathology of salivary duct carcinoma who received 240 mg of nivolumab every 2 weeks. The survival analysis included ORR, OS, and PFS. The correlation between therapeutic outcomes and clinico-histological factors was also examined. Their findings demonstrated 4.2% overall response rate, 1.6 months of median PFS, and 10.7 months of overall survival. Considering the limited efficacy of nivolumab against SGC and the achievement of long-term disease control in some patients, they suggested further research on ICI administration in salivary gland cancer patients (31).

As far as we know, salivary gland cancer is rare, but its heterogeneity due to various histopathological subtypes makes it challenging in response to chemotherapeutic agents and immune checkpoint inhibitors, especially in patients with recurrent or metastatic malignancy. To fill this clinical gap, Nagatani et al. (28) carried out a multicenter phase II trial at nine centers in Japan for investigation of the efficacy and safety of nivolumab administration in patients with platinum-refractory salivary gland carcinoma. Twenty-four patients with a median age of 65.5 years, conducted between March 2018 and January 2022, were enrolled in their study. Every 2 weeks, the enrolled patients received nivolumab 240 mg/body intravenously until progression or unacceptable toxicity. After 32 months of median follow-up, the study results showed 3 months of progression-free survival and 25 months of overall survival in the studied sample. The findings did not add any safety concerns to the previous understanding. The demonstrated study did not meet its primary endpoint of objective response rate, and due to concerns about nivolumab monotherapy, the researchers suggest translational research to increase our understanding of the immune microenvironment over various pathological subtypes, respectively (28).

The combination therapeutic approach of immune checkpoint inhibitors, considering nivolumab and ipilimumab, has received FDA approval for certain malignancies including recurrent NSCLC, melanoma, and hepatocellular carcinoma. Chae et al. (32) conducted a Simon’s two-stage single-institution prospective phase II clinical trial to evaluate the potential of nivolumab and ipilimumab combination in ACC and other salivary gland carcinomas. They enrolled 24 patients, consisting of 19 ACC cases and 5 patients with other subtypes of salivary gland carcinomas. The patients intravenously received nivolumab 240 mg every 2 weeks for 16 weeks and then 480 mg every 4 weeks. Also, the patients intravenously received ipilimumab 1 mg/kg every 6 weeks. The results among ACC patients showed 30 months of median overall survival, 8.3 months of median PFS, and 53% disease control rate. In the cohort of salivary gland tumor, the findings were as follows: 10.4 months of median overall survival, 6.21 months of median progression-free survival, and 40% disease control rate. Interestingly, across the joint cohorts, platelet counts above the median were significantly associated with better overall survival and PFS. Also, in the study, some immune-related toxicities were observed including anemia and lymphocytopenia. In a nutshell, the study showed potential therapeutic benefits in combination therapy of nivolumab and ipilimumab for recurrent or metastatic salivary gland neoplasms (32).

Fayette et al. (27) conducted a phase II single-stage trial with a Fleming design to evaluate the potential of nivolumab in SGC patients who experienced a progressive disease progression over 6 months before enrolling in the study. Ninety-six patients enrolled in the study who were divided into ACC and non-ACC groups received nivolumab for a maximum of 12 months. The researchers analyzed the studied groups’ survival and health-related quality of life. Forty-six cases of ACC and 52 cases of non-ACC SGC were studied with a median follow-up of 29.2 months in the ACC group and 16.9 months in the non-ACC group. According to the study, nivolumab failed to show efficacy in the non-ACC group, but in the ACC cohort, 5.3 months of PFS and 17.2 months of overall survival were demonstrated. The study’s primary endpoint was met in the ACC group, and the limited efficacy of nivolumab in salivary gland carcinoma was supported by the study’s finding (27).

## Conclusion

This meta-analysis shows an association between the progression-free survival of salivary gland cancers as the outcome of interest and the therapeutic potential of PD-1 and PD-L1 inhibitors. According to our results which are supported by previous randomized controlled trials and cohort studies, PD-1 and PD-L1 inhibitors may benefit salivary gland cancer patients in selected cases due to their potential therapeutic effects. The data and evidence gathered by the included studies in this meta-analysis with high heterogeneity led us to recommend that more studies including large-scale cohort studies and randomized controlled trials should be conducted to support further the associations revealed by our findings.

## Data Availability

The original contributions presented in the study are included in the article/supplementary material. Further inquiries can be directed to the corresponding authors.
